# A Focus on the HIV Care Continuum Through the Healthy Young Men’s Cohort Study: Protocol for a Mixed-Methods Study

**DOI:** 10.2196/10738

**Published:** 2019-01-24

**Authors:** Michele D Kipke, Katrina Kubicek, Carolyn F Wong, Yolo Akili Robinson, Ifedayo C Akinyemi, William J Beyer, Wendy Hawkins, Cara E Rice, Eric Layland, Bethany C Bray, Marvin Belzer

**Affiliations:** 1 Division of Research on Children, Youth and Families The Saban Research Institute Children's Hospital Los Angeles Los Angeles, CA United States; 2 The Methodology Center The Pennsylvania State University University Park, PA United States; 3 Adolescent and Young Adult Medicine Children's Hospital Los Angeles Los Angeles, CA United States

**Keywords:** acquired immune deficiency syndrome virus, HIV, cohort study, men who have sex with men

## Abstract

**Background:**

No group is at greater risk for acquiring HIV than young men who have sex with men (YMSM), particularly black or African American (AA) and Hispanic or Latino (L) YMSM living in inner cities, who account for the largest number of new HIV infections each year. Although pre-exposure prophylaxis (PrEP), postexposure prophylaxis (PEP), and treatment as prevention hold enormous promise for changing the course of the epidemic, AA/L-YMSM are the least likely population to be receiving primary health care and HIV prevention/care and are the least likely to be using PrEP and PEP.

**Objective:**

The overarching aim of the Healthy Young Men’s (HYM) cohort study is to conduct longitudinal research with a cohort of AA/L-YMSM to prevent new HIV infections, reduce transmission, and reduce HIV/AIDS-related disparities by focusing on successful engagement in care. Findings from this research will be used to inform the development of new interventions designed to engage AA/L-YMSM in the HIV prevention and care continua.

**Methods:**

Longitudinal research (baseline and follow-up assessments every 6 months for a total of 8 waves of data collection) is ongoing with a new cohort of 450 high-risk AA/L-YMSM in Los Angeles. Participants were recruited using a venue-based and social media sampling design. In addition to self-report surveys, the study protocol includes the collection of urine to assess recent use of illicit drugs and the collection of blood and rectal/throat swabs to test for current sexually transmitted infection (STI)/HIV infection. An additional sample of blood/plasma (10 mL for 4 aliquots and 1 pellet) is also collected and stored in the HYM cohort study biorepository for future research. By design, we recruited 400 HIV-negative participants and 50 HIV-positive (HIV+) participants. This mixed-methods study design includes collection and triangulated analysis of quantitative, qualitative, and biological measures (ie, drug use, STI/HIV testing, and adherence to antiretroviral therapy among HIV+ participants) at baseline and every 6 months. The HYM cohort study will provide a platform from which new and emerging biomedical prevention strategies (eg, PrEP, rectal microbicides, and PEP) and other HIV prevention and care engagement interventions can be developed and evaluated with AA/L-YMSM.

**Results:**

To date, all participants in the HYM cohort study have been recruited and baseline assessment has been conducted.

**Conclusions:**

The findings from this research will be used to inform the development of new and/or adaptation of existing evidence-based HIV prevention interventions and interventions designed to engage this population in the HIV prevention and care continua.

**International Registered Report Identifier (IRRID):**

DERR1-10.2196/10738

## Introduction

### The HIV Epidemic and Young Men Who Have Sex With Men: Correlates and Risk

In this third decade of the HIV epidemic, we continue to see 50,000 new infections annually in the United States, with the highest rate of diagnosed HIV infection among adolescents and young adults (approximately 25%) [1,2]. No group is at a greater risk for acquiring HIV than young men who have sex with men (YMSM), particularly black or African American (AA) and Hispanic or Latino (L) YMSM living in inner cities, who account for the largest number of new HIV infections each year [3,4]. In 2016, AA men who have sex with men (MSM) accounted for 25% of the new HIV diagnoses and 38% of new diagnoses among all gay and bisexual men in the United States. Among AA MSM testing positive in 2016, 36% were aged between 13 and 24 years and 39% were aged between 25 and 34 years [5]. L-MSM experience similar disparities in HIV rates. Between 2000 and 2014, diagnoses among all L-MSM gay and bisexual men increased by 13%; diagnoses among L-YMSM increased by 16% during this same period [6].

Risk factors associated with HIV and other sexually transmitted infections (STIs) among YMSM include alcohol misuse, illicit drug use, involvement in condomless anal sex, and mental health problems, including depression and anxiety [7-12]. However, there is an emerging literature that suggests that AA-YMSM have a unique risk profile, that is, they are less likely than white and L-YMSM to report binge alcohol use and illicit drug use and yet, they experience high levels of racism, discrimination, and stigma, and these experiences, in turn, put AA-YMSM at increased risk for internalized homophobia, maladaptive coping, and/or mental health problems [13,14]. Poor mental health in turn has been found to put AA-YMSM at increased risk for illicit drug use and sexual risk taking [15]. With respect to L-YMSM, they too appear to have their own, unique risk profile, that is, many have come of age in a culture with a strong emphasis on traditional gender roles, family, and having children [16]. Within this context, sociocultural factors such as community connectedness, social support, adherence to cultural values for sex, sexual discomfort (eg, feeling embarrassed or not being able to speak about sexual matters), and self-efficacy to discuss sexual matters significantly predict illicit drug use and sexual risk taking [17-19]. Our research has found that differences in religious experiences, internalized and community homophobia, and identification/disclosure increase L-YMSM’s distress [20].

### HIV Prevention and the HIV Care Continuum Among Young African American and Latino Men Who Have Sex With Men

Despite considerable risk and high rates of new infection among AA-YMSM and L-YMSM, today there is greater hope than ever before that we can change the course of the HIV epidemic given a number of biomedical approaches to prevention that leverage the use of antiretroviral therapy (ART), including pre-exposure prophylaxis (PrEP) and postexposure prophylaxis (PEP). PrEP has enormous promise to limit HIV acquisition, with more than 90% efficacy among those with high adherence. Although awareness of and knowledge about PrEP is quite high among YMSM, uptake is extremely low, especially among AA-YMSM and L-YMSM [21]. National data indicate that about 77,000 people are using PrEP, the majority being white and above the age of 30 years [22]. This low uptake points to social and structural determinants, such as poor access to care and financial barriers related to other needs including food and shelter [21,23]. There is growing evidence demonstrating that although YMSM are generally aware of and have knowledge about PrEP, they are significantly less likely than adult MSM to have ever used PrEP [24,25]. Moreover, among YMSM, AA-YMSM, and L-YMSM are the least likely to have ever used PrEP [26].

**Figure 1:**
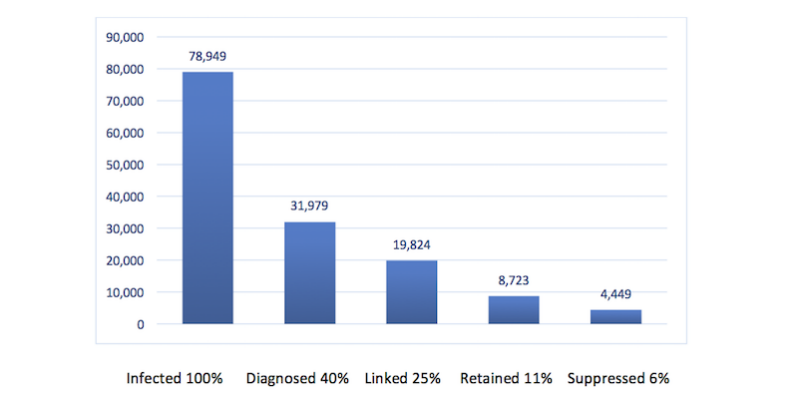
Cascade of care in HIV infected youth in the United States (from Zanoni & Mayer [30]).

Early diagnosis of HIV and timely linkage to and retention in care are vital to survival and quality of life. HIV-positive (HIV+) individuals who adhere to ART exhibit slower disease progression, fewer HIV comorbidities, improved overall health outcomes, and less transmission to partners [27]. Unfortunately, there is growing evidence that HIV+ young people are significantly less likely than HIV+ adults to be linked to and retained in care, to initiate ART, and to experience viral suppression [28-30]. Figure 1 reflects the estimated cascade of care in HIV+ youth (ages 13-29 years) in the United States [29]. As reported by Ryscavage et al, AA youth in care were found to have the lowest probability of viral suppression at 6 months and the highest predicted probability of viral rebound, compared with AA adults, non-AA youth, and non-AA adults [30]. In general, it has been shown that HIV is diagnosed at a later stage among Latinos and that these patients have lower CD4 cell counts, higher HIV RNA levels, more AIDS-defining opportunistic infections, and longer hospital stays than whites [31]. Latinos have also been shown to have significant delays in initiation of HIV care. Reasons for delay of care include lack of access to transportation, being too sick to go to the doctor, and having 1 or more competing needs on expenditures, such as rent and food costs [32].

Many of the HIV disparities that currently exist relate to different patterns in HIV testing, linkage to care, and engagement/retention in care [33,34]. Christopoulos et al concluded that racial disparities in HIV outcomes persist among MSM in large part because of different patterns of engagement in care [35]. Lack of insurance and patient mistrust of health care/providers may also play a key role in this lack of engagement in care. In addition, Christopoulous et al argued that limited research has been conducted to better understand barriers to engagement and retention in care among MSM in general and YMSM in particular. Moreover, they concluded that there is a dearth of research on culturally relevant strategies designed to engage AA-MSM and L-MSM in HIV care, especially AA-YMSM and L-YMSM [35].

### The Healthy Young Men’s Cohort Study: Opportunities to Turn the Curve of the HIV Epidemic

Given these multiple factors and opportunities, the Healthy Young Men’s (HYM) cohort study was designed to provide rich data to help further understand and characterize AA-YMSM and L-YMSM’s engagement in the HIV continuum of care and prevention, including their use of HIV testing services and access to and use of HIV prevention/treatment services. The HYM cohort study was designed to inform the development of developmentally appropriate and culturally relevant interventions addressing the many risk factors that serve as barriers to accessing needed HIV prevention and care services.

### Overarching Goal and Specific Aims

The overarching goal of the HYM cohort study is to conduct longitudinal research with a large and diverse cohort of AA-YMSM and L-YMSM to (1) characterize risk in this population and (2) longitudinally examine transitions and associated risk with transitions related to illicit drug use (eg, from nonillicit drug use to illicit drug use), sexual risk (from low to higher risk behaviors), and STI/HIV infection. Moreover, a primary aim of the study is to inform the development of age-appropriate and culturally relevant interventions that help prevent new HIV infections, reduce transmission, and reduce HIV/AIDS-related disparities. A specific focus is on successful engagement, linkage, and retention in care. Building on the HIV continua of care and prevention paradigm (ie, seek, test, treat, and retain in care) and the syndemic theory of risk [8,36-38], this research addresses 4 overarching research questions: (1) Why do some HIV-negative (HIV−) AA/L-YMSM seroconvert (and not others) and how do we more effectively prevent new infections in this population? (2) How can we more effectively engage AA/L-YMSM in all forms of care, including primary care, HIV testing, HIV prevention, and HIV/AIDS treatment services if HIV+? (3) How can we increase demand/uptake of PrEP and PEP as a prevention strategy in this population? and (4) How do we prevent transmission and achieve disease-free survival by achieving viral suppression in this population? The specific aims are as follows:

- Specific aim 1: Better understand and operationally define what linkage, engagement, and retention to care (both primary health and HIV/AIDS treatment) and PrEP/ART adherence mean to HIV− and HIV+ AA-YMSM and L-YMSM. In addition, use these data to inform the development of new assessment tools for future intervention research.
- Specific aim 2: Characterize and monitor over time AA/L-YMSM’s (1) use of alcohol and illicit drugs; (2) utilization of HIV testing and prevention services; (3) incidence of HIV and STIs; (4) insurance status and access to health care services, including primary care and HIV/AIDS treatment services; (5) engagement in and utilization of health care and HIV/AIDS treatment services; (6) retention in HIV/AIDS care and adherence to ART; and (7) utilization of biomedical interventions, such as PrEP and PEP. A component of this specific aim will include the procurement of biological specimens and an annual HIV viral load (VL) test for each member of the cohort. The goal is to be able to query specimens for biological evidence of adherence, potential markers of increased infectious susceptibility (eg, human leukocyte antigen typing and whole virus sequencing), and/or variability in disease progression rates.
- Specific aim 3: Identify barriers/facilitators of engagement along the HIV continua of care and prevention, including HIV testing and biomedical prevention, care engagement and retention, and adherence to ART. The finding from this research will be used to identify risk/protective influences, including structural and social barriers/facilitators, which we hope to use in future studies to design new prevention interventions targeted to this population, and/or inform the adaptations/further refinement of existing evidence-based interventions to ensure they are developmentally appropriate and culturally relevant for AA-YMSM and L-YMSM.

### Theoretical Model and Conceptual Framework

#### Syndemic Theory

The syndemic theory has increasingly been used to explain MSM and YMSM of colors’ involvement in HIV risk-taking behaviors [8,36-40]. A syndemic is defined as “two or more afflictions, interacting synergistically, contributing to excess burden of disease in a population.” [41]. Syndemic theory posits that a constellation of health problems, including drug use and alcohol misuse, depression, sexual compulsiveness, and intimate partner violence, accumulates across a life span, with each condition potentially amplifying the negative impact of other health problems. For AA/L-YMSM, multiple and overlapping forms of risk—racism, discrimination, and homophobia—correlate with negative health impacts [8,42]. Our previous research has found that AA/L-YMSM experience the highest rates of risk factors as framed by the syndemic theory, including drug use, mental health problems (such as depression), and intimate partner violence. AA-YMSM also experience higher levels of internalized homophobia, which in turn is a strong predictor of sexual risk behaviors. In addition, experiences of racism, homophobia, and violence have been found to be significantly associated with illicit drug use, alcohol misuse, and involvement in HIV sexual risk behaviors [15,43]. The HYM cohort study examines syndemic risk factors as predictors of HIV infection among AA/L-YMSM as well as engagement in care, including HIV prevention, testing and treatment, and adherence to ART.

#### Engagement in the HIV Care Continuum

Successful engagement in care, both primary care for HIV– and HIV care for HIV+ individuals, is now considered essential to achieving critical outcomes required to ensure disease-free survival and to ultimately end the HIV/AIDS epidemic [44]. Under the Affordable Care Act, linkage to HIV testing (as well as affordable insurance) may serve as a critical point of care within the health care system. For those who test HIV+, early diagnosis and linkage to HIV/AIDS care are essential, and yet it is now very clear that *engagement in care* is a complex construct that is perhaps best represented along a continuum of engagement. Moreover, it is also clear that adherence to ART is more likely to occur if individuals are engaged in their own care. Cheever argues that a “person living with HIV may go through several stages and may also return to earlier stages along this continuum throughout his/her life” [45].

**Figure 2:**
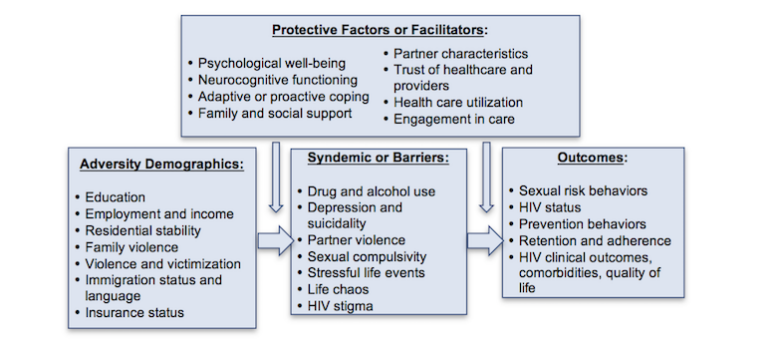
The Healthy Young Men’s cohort study conceptual model.

Mugavero et al developed a framework with 7 steps along a continuum of HIV service delivery, ranging from not in care/unaware of HIV status, to diagnosis (aware of HIV status), linkage to care (receiving some medical care but not HIV care; entered HIV care but lost to follow-up; in/out of HIV care or infrequent user), retention in care (fully engaged in HIV care), and adherence to ART with the goal of VL suppression [44]. We believe this framework provides a more nuanced understanding of engagement. In addition, we believe that different types of barriers and facilitators are important determinants of engagement along this continuum. On the basis of the literature as well as our own research conducted with YMSM, we firmly believe that YMSM of color experience a unique set of challenges to engagement that are developmentally and culturally defined. Figure 2 provides a conceptual framework and analytical plan for our proposed research. We hypothesize that specific demographic and syndemic risk factors/barriers put AA/L-YMSM at significantly greater risk for HIV infection, poor retention in care and poor adherence to ART, and consequently, poor HIV-related clinical outcomes and continued HIV transmission. We also hypothesize that protective/facilitator factors mediate and/or moderate this risk. The collection of longitudinal data over 4 years with a large cohort of AA/L-YMSM (eg, who are both HIV– and HIV+, in and out of care, adherent, and not adherent) allows us to examine trajectories along this continuum and identify predictors of who is and is not engaged/retained in care at each step along the continuum, and why.

The purpose of this study is to describe the HYM cohort study protocol—that is, study design and research methods for this longitudinal study.

## Methods

### Consent and Institutional Review Board Approval

This study has been reviewed and approved by Children’s Hospital Los Angeles’ Institutional Review Board (IRB# 14-00279). All participants were identified, screened for eligibility, and if eligible, invited to participate in the study, as further described below. All participants provided written informed consent during a face-to-face consenting visit. A certificate of confidentiality was obtained from the National Institute on Drug Abuse and a waiver of parental consent/assent was obtained for participants aged 16 to 17 years.

### Study Design

Longitudinal research (baseline and follow-up assessment every 6 months) is in progress with our cohort of 450 AA/L-YMSM in Los Angeles. Participants were recruited using a venue-based and social media sampling design, described below. In addition to self-report surveys, data collection includes urine collection to assess recent use of illicit drugs, rectal and throat swabs to test for gonorrhea and chlamydia, blood draw for syphilis testing, and the additional collection/storage of additional blood (10 mL for 4 aliquots and 1 pellet) and a rectal swab to be stored in the HYM biorepository. This mixed-methods study design includes collection and triangulated analysis of quantitative and biological measures (ie, drug use, STI/HIV testing, and adherence to ART among HIV+ participants) at baseline and every 6 months for a total of 8 waves of data collection. In addition, qualitative substudies are integrated into the study using a modified timeline follow-back approach; these are conducted outside the regular study visits on an as-needed basis. The HYM cohort study has been designed to provide a platform from which new and emerging biomedical prevention strategies (eg, PrEP and PEP) can be developed and evaluated with AA/L-YMSM.

### Study Participants

**Figure 3:**
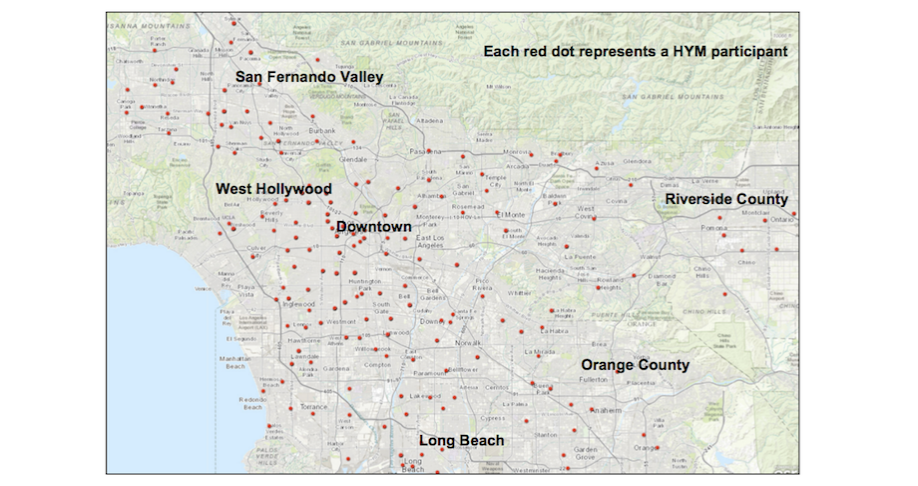
Map indicating where participants of the Healthy Young Men’s cohort study reside.

YMSM youth were eligible to participate in the cohort if they (1) were aged 16 to 24 years; (2) assigned a male sex at birth; (3) self-identified as gay, bisexual, or uncertain about their sexual orientation; (4) reported a sexual encounter with a male within the previous 12 months; (5) self-identified as black/African American, Latino, or multiethnic; and (6) lived in Los Angeles or a surrounding county, with no expectation of moving outside this area for at least 6 months. The recruitment strategy described below resulted in a geographically dispersed cohort recruited from throughout Los Angeles county, as shown in Figure 3.

### Recruitment

#### Identifying Public Venues and Social Media Sites

Formative research was first conducted to identify public venues frequented by AA/L-YMSM. Staff contacted and met venue owners/managers (including HIV test sites and clinic directors) of sites identified for recruitment to explain the study and to obtain permission to conduct activities. Facilitated discussions with the study’s community advisory board (CAB) and youth advisory board (YAB) identified common social media sites and dating apps that are popular among our target population.

#### Recruitment in Public Venues

The recruitment methods in this study matched those used in our previous research with the same target population [46]. Young men were recruited from public venues including bars, coffee houses, parks, and high-traffic street locations where YMSM spend time or *hang out*, social events sponsored by an agency or organization that serves YMSM, and special events such as gay pride festivals and balls.

During sampling events, young men who appeared to meet the study criteria were counted and invited to participate in a screening interview conducted in English or Spanish. A total of 1 or 2 researchers counted and identified young men to be screened and tracked individuals to ensure that young men were not approached multiple times. Young men who met the study criteria received a detailed study description, and contact information was obtained from individuals who expressed interest in participating. Follow-up in-person appointments were scheduled within a week of recruitment to complete the informed consent process and to further explain study participation. For each sampling event, the following data were collected: (1) number of YMSM observed; (2) number of YMSM intercepted; (3) age, race, and county of residence of those screened; (4) reasons for refusal; (5) number of eligible YMSM; and (6) number enrolled.

We originally planned to recruit our cohort of AA/L-YMSM using only this recruitment method. However, during our prerecruitment field observations, the research team noted that few AA/L-YMSM were present at these venues. Discussions with our CAB and YAB indicated the low number of YMSM attending gay-identified venues was a common challenge for outreach; YMSM are simply not attending gay-identified venues as they once did.

#### Recruitment Using Social Media

Given these changes in the community, our team determined that additional recruitment methods were needed to complete recruitment of the cohort within the allotted recruitment time frame. Thus, we partnered with Trialspark, a technology company that supports recruitment for clinical studies/trials using social media sites. The HYM team worked with Trialspark to design social media ads to be placed on sites identified by our YAB including Facebook, Instagram, Grindr, and Jack’d. Through our partnership, Trialspark identified and briefly screened 1371 individuals; of these, 40.11% (550/1371) were identified as eligible. Preliminarily individuals were then independently contacted and rescreened for enrollment by our research team. Young men were also recruited through participant referrals as well as referrals from our partner clinical sites. Table 1 presents the recruitment data for each recruitment method.

### Tracking and Retention

Participants were asked to participate in data collection at baseline and follow-up every 6 months. We acknowledge the complexities of tracking and retaining a young and highly mobile population such as YMSM. Our past experience taught us that the key to retaining youth in a research study is developing trusting relationships between the study team and the research participants. To that end, we adapted a tracking protocol used in previous studies [47], which yielded a 94% retention rate across 2 and a half years and 5 waves of data collection. An essential piece of this protocol is assigning staff to a specific participant, with the goal of maintaining that relationship across the course of the study. Staff turnover is inevitable, and when that occurs, we ensure there is contact between the original staff person, the participant, and the newly assigned staff person to ensure continuity in the relationship.

**Table 1:** Healthy Young Men’s cohort study recruitment and eligibility data.

Recruitment method	Social media, n (%)	Venue/events, n (%)	Participant referrals, n (%)	Clinic, n (%)	Total, n (%)
Approached for screening	1371 (67.64)	544 (26.84)	69 (3.40)	43 (2.12)	2027 (100.00)
Screened for eligibility	690 (50.33)	477 (87.68)	69 (100)	42 (97.67)	1278 (63.05)
Determined to be eligible for study	550 (40.12)	206 (37.87)	64 (92.75)	31 (73.81)	851 (41.98)
Completed baseline survey	350 (63.6)	46 (22.3)	37 (57.8)	19 (61.3)	452 (53.1)

In addition, the protocol also includes gathering tracking and location information including (1) address, (2) phone numbers, (3) email, (4) social media, (5) family/friend contact, and (6) school/work information. Every 6 months, this information is reviewed with the participant and updated as needed. Participants are asked to contact their interviewer monthly (eg, text message, phone call, or SnapChat) in return for a US $7 monthly incentive (an additional US $42 added to their data collection incentive). These check-ins are an opportunity to determine whether the participant needs any resources (eg, food bank and physician referrals) and remind them of any upcoming appointments. The HYM team uses this opportunity to catch up on any events in the young men’s lives and enter field notes as needed. We learned that the HYM participants tend to enjoy these check-ins and share photos with their assigned staff person (eg, prom and weddings) or ask about different services as needed. If the participant fails to make contact after 2 months, the participant’s assigned interviewer uses tracking and location information and/or criminal justice records to make contact. Between baseline and wave 2, only 7% had missed 1 or more check-ins.

### Community Advisory Board and Youth Advisory Board

CABs and YABs have played a critical role in our research conducted with YMSM. The HYM CAB and YAB were developed to help to inform all aspects of the study design, implementation, and interpretation of the study findings. CAB members include policy makers, HIV/AIDS service providers, and community advocates. The YAB comprises members of our target population who were recruited from local clinics serving YMSM. The CAB meets on a bimonthly basis and the YAB meets monthly. Agendas typically include brief study updates, information about new proposals in development and upcoming events, a data presentation on a specific topic or construct, and discussions about how to interpret these data and move them to the next stage. Our CAB assisted in developing community forums, coauthored peer-reviewed papers, assisted in outreach, and copresented study results with the HYM team. The YAB has reviewed our data collection tools, provided feedback on proposed interventions, and assisted in outreach efforts at public venues.

### Measures

HYM study participants participate in a self-report survey every 6 months. The survey is administered by their assigned staff person, and questions about more private topics (eg, substance use, sexual behavior, violence) are self-administered using a Web-based survey to provide an additional layer of confidentiality and encourage more honest responses [48,49]. The survey takes approximately 1 and a half hours to complete; special topics are integrated into individual waves when only a single assessment point of data is needed (eg, childhood trauma or mindfulness). At baseline, participants completed a *pre-baseline* assessment, a brief (10-min) survey completed during the informed consent process, and received US $10 for the pre-baseline and an additional US $55 for completing the baseline assessment. Participants can earn up to US $100 at follow-up assessments if they complete each monthly check-in (US $55 for the assessment and US $42 for the check-ins, rounded up to total US $100). A description of the study measures is as follows.

#### Demographic Characteristics

Survey instruments obtain demographic information including age, race/ethnicity, religion residence and residential stability, education/employment, food security/hunger, socioeconomic status, history of foster care and incarceration, and insurance status.

#### Primary Outcome Measures

##### Alcohol, Tobacco, Marijuana, and Illicit Drug Use

Scales from the Monitoring the Future study are used to assess lifetime, past 6-month, and past 30-day illicit drug and alcohol use [50]. The drug list includes marijuana, lysergic acid diethylamide or LSD, phencyclidine (more commonly known as PCP or angel dust), mushrooms, cocaine, crack, methamphetamines, ecstasy, stimulants, heroin, fentanyl, and prescription drugs used without a physician’s order. We also assessed substance use problems using standardized measures including alcohol and marijuana misuse. Participants are asked the location and circumstances during which they use drugs, particularly around the time when they engage in sexual behaviors. We collect urine samples at baseline and every 6 months to test for metabolites of methamphetamines, cocaine, ecstasy, marijuana, and opiates using the Integrated E-Z Split Key Cup II- 5 Panel (Innovacon Laboratories), which can detect drugs from 1 to 4 days after use, except for chronic marijuana use, which can be detected for up to 30 days [51,53]. Screening for fentanyl is also performed.

##### Sexual Risk Behaviors, Partners and HIV Risk, and Protective Behaviors

These are assessed using scales adapted from the EXPLORE study and research we previously conducted with YMSM [52,53]. Participants are asked about their *lifetime and recent sexual experiences* (past 1 and 6 months), including insertive/receptive oral sex, insertive/receptive vaginal sex, and insertive/receptive anal sex. Specifically, participants are asked to report the number of times they engaged in each type of sexual activity and the gender of their partners, each of these types of sexual activity for the *different partner types* (eg, primary, consistent casual, and casual) they might have had in the past 6 months, and the *frequency of condom* use by gender of partner and by sexual activity. This measures *condomless intercourse*. Participants are asked if they have ever and recently (past 6 months) exchanged sex for money, drugs, food, clothes, etc.

##### Condom Use Self-Efficacy

The 15-item Condom Use Self-Efficacy measures condom use self-efficacy using 5-point Likert scale [54,55]. Condom use intention is assessed using a 9-item condom intention scale [56].

##### Partner Demographic and HIV Status

Current partner or partners’demographics including race/ethnicity are age, type (primary, casual, and hookup; if primary open vs monogamous), HIV status and HIV concordance/discordance, and partner’s use of HIV services and ART adherence if HIV+. Sexual compulsivity is also assessed with the 10-item Sexual Compulsivity Scale, which asks respondents to endorse their agreement with statements related to sexually compulsive behavior [57].

##### Sexually Transmitted Infection/HIV Testing and Prevention Behaviors

Participants complete HIV testing using fourth-generation point-of-care rapid whole blood finger-stick HIV test (Alere, Inc, Waltham, MA), an FDA-approved diagnostic measure of HIV-1 p24 antigen and HIV-1/2 antibodies. This test is performed every 6 months. We also use scales from our previous study conducted with YMSM to measure self-reported history of HIV/STI testing and HIV status*.* Participants self-collect rectal and pharyngeal specimens for *Neisseria* *gonorrhea* and *Chlamydia trachomatis* nucleic acid amplification testing at baseline and every 6 months. Syphilis testing is performed using whole blood collected via venipuncture (or fingerstick) using rapid plasma regain and treponemal antibody testing at baseline and every 6 months. Those with positive results meet the on-site HIV test counselor and then are referred to and treated at 1 of our partner clinical sites.

##### Health Care, Linkage, Engagement, and HIV Service Utilization

Health care, linkage, engagement, and HIV service utilization dates are collected every 6 months. Participants are asked questions about their current health status using modified questions from the Youth Risk Behavior Survey [58]. These questions ask about the respondent’s overall health status, the number of days in the last week they have eaten fruits or vegetables, and the number of days in the last week they have exercised. Participants’ access to and use of the health care system was measured using both the Addhealth survey from the National Longitudinal study of Adolescent to Adult Health study and the National Survey of Children’s Health [59]. These measures assess the frequency and type of health practitioner seen in the past 12 months, insurance status, reasons for use or nonuse of health care services in the last year, and comfort in speaking with their doctor about sexual health. Trust/mistrust of the health care system is measured with the Health Care System Distrust Scale, a 10-item scale that assesses perceptions of the health care system [60]. Visual analog scale (VAS) for ART adherence asks participants to consider a specific period (eg, last month) and to estimate the percentage of medication doses taken [61,62]. VAS has moderate correlations with unannounced pill counts and self-reported recall and is widely used to assess medication adherence.

#### Possible Mediating/Moderating Constructs

##### Depression, Mental Health, Spirituality, Well-Being, Optimism, Resilience, and Mindfulness

To assess depression, anxiety, and somatization, we used the 18-item Brief Symptom Inventory (BSI) [63]. The Patient-Reported Outcomes Measurement Information System (PROMIS) depression short scale assessed depressive symptoms during the previous 7 days [64]. PROMIS was administered during baseline, whereas BSI was administered at baseline and in all follow-up waves. Spirituality was found to be an important aspect of young men’s lives in our prior research; thus, we included the spirituality scale, which taps into self-discovery and eco-awareness, 2 of the primary components of spirituality [65]. The 4-item Health as a Value scale measures individuals’ perceived importance of health and well-being. We also assess suicidality and self-injurious behaviors, both current and past histories. Optimism is measured using the 10-item Life Orientation Test-Revised [66], and resilience is measured with the Connor-Davidson Resilience Scale [67]. Mindfulness is measured using the Mindful Attention Awareness Scale, a 15-item scale designed to assess a core characteristic of dispositional mindfulness, namely, receptive awareness of and attention to what is taking place in the present [68].

##### Emotion Regulation and Coping

Emotion regulation and coping data are collected annually. The Difficulties in Emotion Regulation Scale measures participants’ ability to be aware of, understand, and accept their emotions as well their ability to act in desired ways regardless of their emotional state [69]. We assessed a variety of coping strategies participants might use in response to a specific stressor using the Brief COPE [70].

##### Childhood Abuse/Trauma, Internalized Homophobia, Partner Violence, Racism, and Discrimination

Childhood trauma was measured at wave 2 using Bernstein’s Childhood Trauma Questionnaire [71]. Internalized homophobia is assessed using a 4-item measure by Ross and Rosser [72]. Partner violence data are collected annually with the revised Conflict Tactics Scale, which measures violence within the context of intimate relationships and identifies lifetime and past 12-months’ experiences of physical, sexual, and emotional abuse as a victim and perpetrator [73]. Experiences of racism and discrimination are captured using Diaz and Ayala’s 20-item scale that measures lifetime and recent experiences of social discrimination (racism, police brutality, and discrimination due to sexual identity) [74,75]. These data are collected every 6 months.

##### Stressful Life Events and Life Chaos

Stressful life events and life chaos are measured every 6 months. Stressful life events including health-related stress are assessed using a checklist of life events [76]. The scale provides participants with a list of stressful events and asks them if these events occurred during the previous 6 months and their level of stressfulness on a scale from 1 to 10. We also measure life chaos, a construct found to be associated with poor adherence to ART, using the 6-item Life Chaos scale [77].

##### Social Support and Connection to Community

These data are collected every 6 months. The Multidimensional Scale of Perceived Social Support, a 12-item scale, measures perceived social support from family, friends, and partner(s) [78]. Participants’ connection to community—work, school, spiritual, residential, and ethnic—is measured using a 10-item scale developed by our research team and used in our prior research.

### Biological Specimens and Biorepository

A 10-mL EDTA anticoagulated whole blood sample is drawn, and 2 rectal swabs are collected and banked for HIV– participants every 12 months throughout the duration of the study; samples are drawn and banked for HIV+ participants every 6 months. Blood specimens are processed to harvest plasma and a cellular pellet. Plasma is then divided into 4 separate aliquots and stored at −80°C. The red blood cell/buffy coat pellet is harvested and stored for future cellular material and will be made available to investigators for future studies of patient and/or viral genomes. All specimens are entered into a secure, password-protected database noting its position in storage (eg, rack, box, and position) for ease of tracking and retrieval.

## Results

To date, the HYM cohort study sample has been recruited and analyses are being conducted with the baseline data. Assessments will continue every 6 months until the end of this project period, in July 2020.

## Discussion

To date we want to acknowledge:

- Limitations and implications for generalizability of the findings as they are only representative of YMSM of color in the Los Angeles metropolitan area
- Our retention rate of 97% at 12 months
- Nearly 5% of the HIV-seronegative sample have seroconverted since baseline

Importantly, HYM cohort study participants are recruited from communities experiencing many disparities in health care and other social determinants of health (eg, poverty, low education levels, and crime). Therefore, it is important to consider how both positive and negative changes in these disparities over time may facilitate young men’s engagement in HIV care, utilization of prevention strategies, and ultimately predictors of avoiding HIV seroconversion and incident STIs.

## Abbreviations

AA/L-YMSM: black or African American (AA) and Hispanic or Latino (L) YMSM
ART: antiretroviral therapy
CAB: community advisory board
HIV−: HIV negative
HIV+: HIV positive
HYM: Healthy Young Men’s cohort study
MSM: men who have sex with men
PEP: postexposure prophylaxis
PrEP: pre-exposure prophylaxis
PROMIS: Patient-Reported Outcomes Measurement Information System
STI: sexually transmitted infection
VAS: visual analog scale
VL: viral load
YAB: youth advisory board
YMSM: young men who have sex with men
